# Digital COVID-19 surveillance tool for a mass gathering event - a prospective cohort study

**DOI:** 10.1093/eurpub/ckac131.561

**Published:** 2022-10-25

**Authors:** N Hohmuth, A-L Lang, C Remschmidt, R Leistner

**Affiliations:** 1 Data4Life, Potsdam, Germany; 2 Institute of Hygiene and Environmental Medicine, Charité, Berlin, Germany

## Abstract

**Introduction:**

Mass gatherings have been associated with a high risk of SARS-CoV-2 transmission. On-site research can foster knowledge of risk factors for infections and improve risk assessments and precautionary measures at future events. We tested a web-based participatory disease surveillance tool to detect COVID-19 infections at and after an outdoor mass gathering by collecting self-reported COVID-19 symptoms and tests.

**Methods:**

We conducted a digital prospective observational cohort study among fully immunized attendees of a sports event that took place from September 2 to 5, 2021 in Thuringia, Germany. Participants used our study app to report demographic data, COVID-19 tests, symptoms, and their contact behavior. This self-reported data was used to define probable and confirmed COVID-19 cases during the full “study period” (08/12/2021 - 10/31/2021) and within the 14-day “surveillance period” during and after the event, in which the highest likelihood of an event related COVID-19 outbreak could be expected (09/04/2021 - 09/17/2021).

**Results:**

A total of 2,808 of 9,242 (30.4%) event attendees participated in the study. During the study period, 776 symptoms and 5,255 COVID-19 tests were reported in the study app. During the surveillance period, seven PCR positive COVID-19 cases were found to be associated with the event. This translated to an estimated seven-day incidence of ∼125/100,000 cases (95% CI [67.7/100,000, 223/100,000]), which was comparable to the average age-matched incidence in Germany during this time (118.3/100,000).

**Conclusions:**

COVID-19 cases attributable to the mass gathering were comparable to the German-wide age-matched incidence, implicating that our active participatory surveillance tool was able to detect mass gathering related infections. Further studies are needed to evaluate and apply our participatory disease surveillance tool in other mass gathering settings.

**Key messages:**

• Our digital COVID-19 surveillance tool for mass gathering events was easy to implement within the organizational structure of the event and well accepted amongst event attendees.

• Our active participatory surveillance tool was able to detect mass gathering related infections comparable to the Germany-wide incidence.

